# Supplementary figures and images for: Epidemics and local governments in struggling nations: COVID-19 in Lebanon
Source: PLoS One. 2022 Jan 27;17(1):e0262048. doi: 10.1371/journal.pone.0262048 (PMC8794115; doi:10.1371/journal.pone.0262048)

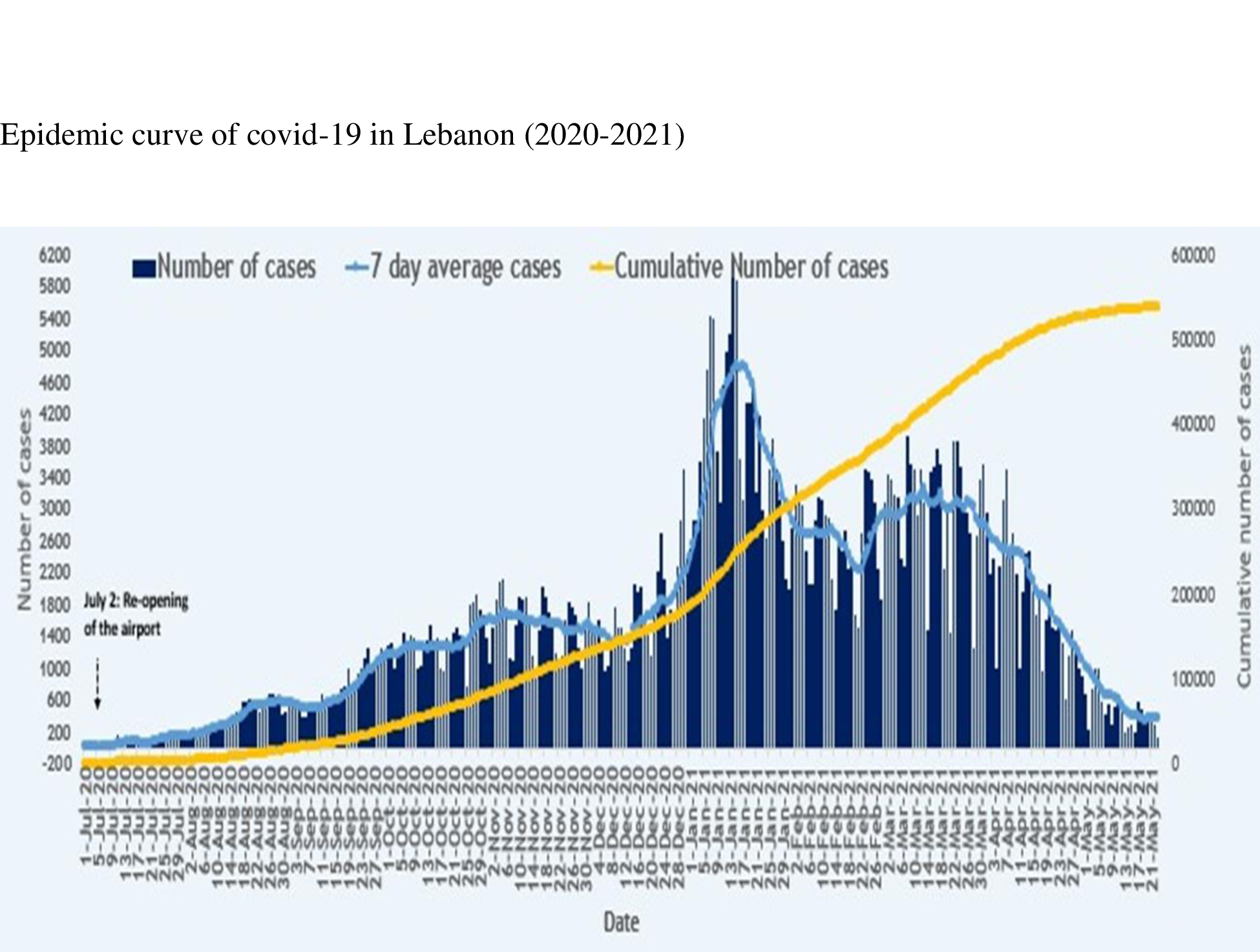

Supplement: S1 Fig — (TIF) [file pone.0262048.s001.tif]
