# Supplementary material for: Epidemics and local governments in struggling nations: COVID-19 in Lebanon
Source: PLoS One. 2022 Jan 27;17(1):e0262048. doi: 10.1371/journal.pone.0262048 (PMC8794115; doi:10.1371/journal.pone.0262048)
Supplement: S1 File — (DOCX) [file pone.0262048.s002.docx]

Basic mandates of municipalities as requested by the government

**Basic mandates of municipalities during COVID-19 as entitled by the government**

- Successful formation of local crisis taskforce
- Raising awareness on COVID-19 transmissions and preventive measures (media platforms, fliers, street tours)
- Sterilizing the streets, cars, common sections in buildings, and places of worship
- Assigning a dedicated helpline
- Fixing checkpoints on entry points and closing exits
- Distributing food rations and in-kind donations
- Distributing masks and disinfecting supplies
- Ensuring adherence of the community to lockdown measures
- Deploying the municipal police to enforce governmental measures, prohibit gatherings, close institutions, and fine people who violate the law
- Sorting and side-treating the garbage of infected patients

**Reference**

Regional Preparedness and Response Plan for COVID-19 – final draft version. (2020). *Coronavirus Disease 2019 (COVID-2019) Health Strategic Preparedness and Response Plan*. Retrieved from: [Leb nCoV Strategic Response Plan MARCH 2020-converted.pdf (moph.gov.lb)](https://www.moph.gov.lb/userfiles/files/News/Leb%20nCoV%20Strategic%20Response%20Plan%20MARCH%202020-converted.pdf)
