# Supplementary material for: Epidemics and local governments in struggling nations: COVID-19 in Lebanon
Source: PLoS One. 2022 Jan 27;17(1):e0262048. doi: 10.1371/journal.pone.0262048 (PMC8794115; doi:10.1371/journal.pone.0262048)
Supplement: S2 File — (DOCX) [file pone.0262048.s003.docx]

**Beyt El-Faqs Municipality**

### Brief Insight on Beyt El-Faqs

Beyt el Faqs is a small town located in Miniyeh-Danniyeh District in North Lebanon with an area of 0.660 km2. The number of inhabitants in this town does not exceed 4000 people. It is distinguished by agriculture, poetry, and high level of education with a large number of doctoral holders, engineers and technicians. The municipality of Beit Al-Faqs was established in 1972 and it consists of 12 members. Beyt Al-Faqs has Al-Nahda primary healthcare center which was established in 1965.

- **Data Collection Process**

Three stakeholders were purposively selected from this municipality: an official, stakeholder1, and stakeholder2. One of my family members connected me with the two key stakeholders, who in return linked me to the official. Each interview took between 30 and 40 minutes.

- **Beyt El-Faqs during Covid-19 pandemic**

*Preparedness of Beyt El-Faqs*

The Ministry of Interior entitled all municipalities to form a crisis cell to manage the pandemic. Since the mayor of Beyt El-Faqs is residing outside the country, the crisis cell has been formed of only four civil society volunteers, with two of them contracted the virus early and got motivated to help other people. Fortunately, this village was privileged to have the head of COVID-19 Unit at one of the largest tertiary hospitals in Lebanon a descendant of this village, who lent a hand and was a credible source of information throughout this crisis. The Covid-19 pandemic has first hit Beyt-el Faqs in August 2020. The cumulative number of Covid-19 cases in Beit El Faqs to the time of interview (13/11/2020) exceeded 250 cases with five deaths in total.

*Initiatives taken by the crisis cell*

The civil society volunteers have undertaken several exemplary initiatives to mitigate the impact of this crisis on their community. On the awareness level, stakeholder1 mentioned: *“We were engaged in raising awareness on social media platforms and through guided tours to shops, cafes, and mosques to urge them to take full precautions. We used speakers in the streets to raise awareness”*. At the beginning of the pandemic, they were able to rapidly assess the cases, trace their contacts, and reach the index case. The first initiative started by raising funds from the well-offs in the village. They were able to allocate a fully equipped room in the PHC to manage mild to moderate cases, distributed in-kind donations (food) to all infected patients with financial difficulties and manage all cases on their expense . In this regard, they provided full medical support and treatment to all COVID-19 patients through home care support with rigorous follow up on daily basis. In addition, they have covered all hospital expenses and transferred critical patients with a fully equipped ambulance. In addition, they have bought oximeters, thermometers, and COVID-19 medications (32 Remdesivir ampoules, antibiotics, supplements, and four plasma units) to manage all cases for free. Also, one of the well-offs donated an oxygen respirator. In case of death, they have a special team for mortgage, and they follow international recommendations to complete this process. On these initiatives, stakeholder2 reflected: *“We assess the cases every day and send medical reports of critical cases to the pulmonary physician who decides on the case. We were able to spare a lot of hospital admissions and save a lot of money through our management”*

*Barriers faced by the crisis cell*

Despite the distinctive achievements, the civil society volunteers were faced with several challenges. Seven major barriers were identified: *Patronage, favoritism, and unauthorized municipality, absence of the central government, lack of awareness and stigma, impeded accessibility to healthcare services, lack of resources, economic collapse and poverty*, and *the* *ignorance of religious leaders.*

The absence of authority is manifested by the lack of municipal police, the inactive municipality which lacks an action-oriented vision, and the absence of the mayor. In this perspective, stakeholder2 stated: *“The key official is living outside the country. He is not effective, and he does not engage in crisis cell meetings. As for another official, he does not present to regular meetings except for formal ones. The only initiative taken by the municipality is allocating 15 million LBP which are insufficient to treat a single COVID-19 patient”.* Consistent with stakeholder2 reporting, stakeholder1 emphasized, *“The absence of a key official impeded us from taking prompt decisions from the side of the municipality. Also, we do not have municipal police because we do not have money to pay for them. Therefore, we faced a lot of resistance from the community who refused to abide to our decisions”.* Alongside, stakeholder2 highlighted that patronage and favoritism are prevailing in the village causing discord and lack of coordination.

The unauthorized municipal council is coupled by the absence of the central government and the lack of coordination with the municipality since the beginning of the crisis. Besides, the interviewed stakeholders complained of the inconsistent implementation of lockdown decisions among different regions, and the discord between the district’s physician and the village representatives. In this regard, t stakeholder1 stated: *‘All measures taken are individual initiatives. “The central government is completely absent and the lack of consistency in control measures taken between villages pose a major barrier against the effective implementation of these measures. When this municipality decided to lockdown the villages both from the entry and exit after the increase in the number of confirmed covid-19 cases, this measure has provoked neighboring village to close the roads on the residents of Beyt El Faqs because it was taken with high sensitivity and it was a personal decision rather than a ministerial one”.* He presumed: “*Another major problem is the discord with the District’s physician who was completely absent and not responding to our needs*”. Stakeholder2 added, “when the Ministry of Interior imposed a lockdown for seven days, the residents adhered to this resolution for the first 2 hours only”.

Regarding the cultural barriers, stigma, stereotyping, and lack of awareness were among the most stressed hurdles. On these barriers, the official, stakeholder1, and stakeholder2, respectively disclosed, *“Rumors were prevailing, and people were getting information from the internet. They considered it a conspiracy from the government to earn more money on their expense, and this situation was further exacerbated if mistakes occur in PCR results”. “There was lack of awareness, and people were not believing in the presence of the virus”. “Stigma was associated with this disease and infected people refrained from disclosing their infection and reporting to the crisis cell for management or hospital transfer”.* To avoid the stigma associated with PCR testing, the crisis cell volunteers opted to diagnose the cases based on their symptoms and CT scan results. Besides, listing the names of cases on Facebook to facilitate tracing their contacts created a stigmatized environment and people got outraged. Thus, they stopped this strategy and resorted to WhatsApp groups.

As for the impeded accessibility to healthcare services, stakeholder1 emphasized: *“There are no available beds in governmental hospitals, private hospitals are overpriced, and all hospitals are far-distanced. We also find difficulty in transporting patients by equipped ambulances”.* Alongside, stakeholder2 accentuated the negative attitudes associated with the care provided by governmental hospitals. He also accused the government of its lack of support to PHCs which are severely underequipped and lack experienced and enough healthcare professionals. He added: *“the civil society alone cannot fight corona solely without the presence of a complete medical team”.*

Another fundamental barrier is the severe shortage in financial, human, and other resources. The government did not pay the municipality’s receivables since long time. This is coupled by a shortage in medical utensils and sanitary supplies. *“the central government fell short of meeting the communities’ needs”*, stakeholder1 commented.

On top of all the barriers, the economic collapse aggravated the repercussions of the pandemic. On the other side, the economic crisis has further burdened people who earn daily wages, increased poverty rates and caused agricultural recession. Due to the multiple crises in Lebanon, including the pandemic, people are suffering from several mental illnesses including stress, depression, anger, and depression.

Stakeholder2 raised an important issue related to the ignorance of some religious leaders during this crisis which affected the compliance of the community towards the preventive measures. He reported that religious leaders were skeptical regarding the presence of COVID-19, and they were inciting people to disbelieve in it. People visiting the mosques were not putting masks and were not abiding to any preventive measure.

**Table. Facilitators, barriers , and outcomes of Beyt El-Faqs municipality**

| Municipality name: Beyt EL Faqs | | District: Minnieh-Danniyeh | | Governorate: North |
| --- | --- | --- | --- | --- |
| Stakeholders: Official, stakeholder1, stakeholder2 | | | | |
| *Facilitators* | ***Barriers*** | | ***Outcomes*** | |
| Prompt setting of a comprehensive emergency plan that is capable to manage 200 new potential patients | Stigma, discrimination, and increased mental illnesses | | - - - Fulfilling of basic mandates (raising awareness on social media and through guided tours, distributing masks, hand sanitizers, sterilizing homes, mosques, and shops)     - Closing the entries and exits of the village without ministerial decision - Distribution of food donations to houses of all infected patients with financial difficulties - Supply of medical equipment (oxygen respirators, oximeters) - Provision of fully equipped ambulances - Home-care management of COVID-19 patients on daily basis and rigorous follow up - free-of-charge provision of COVID-19 and chronic medications - Free-of-charge PCR testing and full hospital coverage to COVID-19 patients - Proper burial of COVD-19 patients consistent with international recommendations | |
| Networking with resourceful healthcare providers | Prevailing patronage, favoritism, and inactive municipal members | |  |  |
| Active civil society volunteers who overtook the role of municipalities | Discord between the municipality and District’s physician | |  |  |
| Increased donations from immigrants and well-offs in the community | Severe shortage in human and financial resources | |  |  |
| Increased credibility and trust in civil society | Lack of coordination, authority, and support from the central government | |  |  |
| Improved self-awareness and health literacy among people | Inconsistency in implementing lockdown measures across neighboring villages | |  |  |
|  | Lack of awareness, spread of fake news, reliance on faith and poor compliance of the community to preventive measures | |  |  |
|  | Delayed reporting of cases | |  |  |
|  | Ignorance of some religious leaders who advocate for non-compliance to preventive measure | |  |  |
|  | Impeded accessibility to healthcare services (congested far-distanced hospitals, over-priced private hospitals, shortage in experienced healthcare providers, and underequipped PHCs) | |  |  |
|  | Agricultural recession | |  |  |
|  | Economic collapse and prevailing poverty | |  |  |
|  | Absence of municipal police | |  | |
