# Supplementary material for: Epidemics and local governments in struggling nations: COVID-19 in Lebanon
Source: PLoS One. 2022 Jan 27;17(1):e0262048. doi: 10.1371/journal.pone.0262048 (PMC8794115; doi:10.1371/journal.pone.0262048)
Supplement: S3 File — (DOCX) [file pone.0262048.s004.docx]

**Michmich Municipality**

- **Brief insight on Michmich:**

Michmich is one of the largest villages in Akkar governorate that extends over an area of 37 km and hosts around 25000 inhabitants, most of them are of Sunni religion. However, with time thousands of the inhabitants have migrated overseas or sought work in neighboring cities, thus the actual number of inhabitants does not exceed 20000 persons. Michmich, like other regions in Akkar, buckles under extreme poverty and is living in a state of deprivation and neglect from the government. Michmich has a predominantly agarian society and is distinguished by its plentiful agricultural lands.

- **Data Collection Process**

For this municipality, three stakeholders were interviewed: official, stakeholder1, and stakeholder2. Before heading to interviews, I have collected background data on this village from a friend of mine who is a resident of this village and from the municipality Facebook page. Then I opted to interview the official. Through the snowballing technique, the official praised the role of the PHC during the pandemic. Subsequently, stakeholder2 stressed the indispensable role of the international Non-governmental Organization (NGO) that cover Akkar region and provided substantial support to the PHC in Michmich.

- **Michmich amid COVID-19 pandemic**

*Preparedness of Michmich*

Aligning with the ministerial decision, Michmich municipality formed a crisis cell composed of municipal members, head of PHC, and community leaders. They set a short-term preparedness plan that focuses on raising awareness, disinfection, and detecting cases. However, soon after the municipality was faced with severe shortage in resources, the plan was suspended, and the municipality surrendered to the fiercely pandemic. As per the IMPACT platform, the cumulative number of COVID-19 cases in Michmich as of April 10, 2021 is 221.

*Initiatives taken by the municipality*

Concerning the initiatives taken by the municipality, the official said that they raised awareness through posters and guided tours, disinfected schools and called for full hygienic precautions, however all their efforts went askew. On the other side, stakeholder2 iterated that the municipality gave up quickly without taking any attempt to resolve the problem in this village. In contrast, the stakeholder1 stressed the positive contribution of the PHC in collaboration with the NGO that filled some of the gaps through their initiatives. The PHC assessed and managed a lot of COVID-19 cases with the limited resources they have. The NGO also subsidized the PHC services so people can have medical consultation for only 3000 LBP.

*Barriers facing the municipality*

Seven major themes were generated as barriers that impeded the municipality from responding properly: *Herd immunity and underreporting,* *stigma and lack of awareness, favouritism, political inducements and inactive municipality, shortage in resources, absence of the central government, impeded accessibility to hospitals, economic collapse and mounting poverty*

The experience of Michmich with COVID-19 pandemic was described as harsh and unsuccessful. *“95% of cases in Michmich are not reported and we are relying on herd immunity”*, the official pinpointed. Besides, stakeholder1 revealed that all COVID-19 cases managed at the PHC were not reported to the MOPH because they didn’t do the PCR and were identified from their symptoms. He added *“I bet if the government does PCR, they will detect more than 20000 cases”.* He presumed, *“when I and my family got infected, I am the only one who did the PCR, while my family members were considered ill without testing”.* He also stated, *“the case of Michmich is a sample of what is happening in Akkar region”.* There is no accurate estimation of COVID-19 cases in Michmich, simply because people refuse to do PCR testing. Upon asking about the increased death tolls shown on their Facebook page, the official and stakeholder1 confirmed that most of the cases are COVID-19, yet not confirmed by PCR. Besides, they bury them without any special precaution.

Upon interrogating the cause of PCR refusal, the official stated, *“People consider corona as an enemy that wants to break their social ties and damage their society”. “They believe that God will protect them, and they don’t believe in anything else”.* People are reluctant to do PCR because they fear social labelling, discord, and stigma. However, financial hardship also deterred people from doing the screening test, the official reported. This coincides with the reporting of stakeholder2 who declared that stigma is prevailing in Michmich pinpointing to an incident that happened with him. He narrated, *“ I used to check on COVID-19 patients at home and provide them with medical care. Two patients refused to enter their home because I was wearing PPE claiming that I am exposing them, and this brings them stigma”.* He presumed *“When I tell someone that he has symptoms of COVID-19, he attempts to attack me”.* Aside from stigma and bullying, financial hardship also hampered people from doing the PCR, the stakeholders reflected.

The official declared that there is a dereliction from the municipality side and that the municipality is part of the corrupted system. Aligning with this confession, stakeholder1 and the stakeholder2 referred the failure of the municipality due to favoritism and unqualified municipal members who lack managerial roles and the experience in this domain. Additionally, the mayor can impose his authority on his allies and not on his proponents, stakeholder2 added. On this aspect, the official mentioned “A powerful municipality needs competent, loyal members to steer its wheel”.

Nonetheless, people have social obligations, and they purposively attend large social occasions. “*Personally, yesterday, I, the 70 years old man, attended a wedding for my cousin with more than three hundred attendees, but I was putting a mask”,* the official continued. *“Even when the municipality distributed masks to people, they put it on their heads or in their pockets”.* However, stakeholder1 presumed that none of the COVID-19 cases were biologically confirmed, they were assessed based on their symptoms. Other initiatives included raising awareness and preventing people from accessing the PHC services if they were not abiding to the preventive measures. stakeholder1 also revealed that he used to provide home care for COVID-19 patients upon request and people were very satisfied by the services provided.

The official referred to the dire unmet needs of the municipality and the neglection of the central government saying, *“we don’t have the capacity to screen for the actual cases in the village”.* stakeholder1 reiterated the severe financial constraints in light of the economic collapse, the lack of support from the government, and the absence of the authority of the security forces. Regarding the harsh financial conditions, stakeholder1 commented *“People don’t have money to buy a mask, and they use the same mask several days which increases their risk of contracting COVID-19”*. On the other hand, the municipality is constrained by the severe shortage of trained human resources and essential preventive utensils (example, masks, PPEs).

All stakeholders commented on the dereliction of the central government given that the lockdown decisions were not compounded by legal provision that hold people accountable for their acts. Neither the military nor the municipality can force people to stop the wedding because of the absence of legislation, stakeholder2 clarified. He also added *“the security forces are hesitant to enforce the lockdown and detain people in the police station because of their limited capacity and their fear of contracting COVID-19”.* Besides, the official mentioned, *“despite the small number of cases reported, MOPH deterred from recognizing this village and doing screening for it”.* Alongside, stakeholder2 blamed the government for not doing PCR for needy people and considered its role absent. Other barriers were shaped by the lack of awareness, stigma and lack of trust in the government, which is corrupted, stakeholder1 mentioned. Besides, the inability of the municipality to designate an isolation center mainly stems from their limited capabilities and due to political inducements, stakeholder1 iterated.

| Municipality name: Michmich | | District: Akkar | | Governorate: North |
| --- | --- | --- | --- | --- |
| Stakeholders: Official, stakeholder1, stakeholder2 | | | | |
| *Facilitators* | ***Barriers*** | | ***Outcomes*** | |
| Collaboration with NGO and Red Cross | Stigma, stereotyping and denial of COVID-19 presence | | - - - Fulfilling of basic mandates (raising awareness on social media and through guided tours, enforcement of hygienic precautions in schools) - Provision of subsidized medical consultation in PHCs | |
| Increased donations from immigrants and well-offs in the community | Herd immunity and underreporting | |  |  |
| Heightened sense of empathy among people | Favoritism, tribal clashes, and political inducements for the municipal position | |  |  |
| Increased job opportunities for some people (ex. nurses, para medicals) | Incompetent and unqualified crisis cell members who surrendered | |  |  |
| Effective contribution of PHCs | Strong community resistance due to lack of awareness leading to increased death tolls | |  |  |
|  | Severe shortage in all resources (human, financial , and relief supplies) | |  |  |
|  | Collapsing economy, prevailing poverty, and harsh financial challenges affecting middle-income people the most | |  |  |
|  | Absence of the central government and underperformance of security forces | |  |  |
|  | Overwhelmed hospitals that cannot accommodate the mounting numbers of cases | |  |  |
|  | Absence of legislation to enforce lockdown decisions by the juridiciary | |  |  |
|  | Lack of isolation center | |  |  |
|  | Lack of comprehensive preparedness plan | |  |  |

Table. Facilitators, Barriers, and Outcomes of Michmich Municipality
