# Supplementary material for: Epidemics and local governments in struggling nations: COVID-19 in Lebanon
Source: PLoS One. 2022 Jan 27;17(1):e0262048. doi: 10.1371/journal.pone.0262048 (PMC8794115; doi:10.1371/journal.pone.0262048)
Supplement: S4 File — (DOCX) [file pone.0262048.s005.docx]

**Amioun Municipality**

- **Brief insight on Amioun municipality**

Amioun is the administrative center of Koura district located in the North Governorate. It extends over an area of It is located on a range of beautiful hills midway between the mountains and the sea. Its total number of populations is around 10,000 inhabitants, with most of them being Greek Orthodox. Amioun is characterized by a substantial number of churches, historical sites, its olive trees, and the high-grade olive oil.

- **Data Collection process:**

Amioun municipality has been prominently highlighted on media as a pioneer among its rivals. I have retrieved the contact number of a key official from the Ministry of Interior and Municipalities. In the interview, he referred to the active role of stakehoder1 who visits COVID-19 patients on daily basis, so I asked him to provide me with her contact number. Consequently, through the same snowballing technique, stakeholder1 pinpointed that there stakeholder2 who also contributed effectively to the community. After I explained the purpose of the study, I got their oral consent, and interviews were conducted. Interviews lasted between 25 and 40 minutes on average.

- **Amioun amid COVID-19 pandemic**

*Preparedness of Amioun municipality*

Following the ministerial decision that called the municipalities to respond quickly to the pandemic, Amioun municipality formed a crisis cell and set a preparedness plan to tackle different levels (medical, social, and fundraising). Then, a smaller active cell of five members was initiated, including official, a nurse, and assistants, and tasks were distributed among the members. The municipality also collaborated with the University of Balamand and Batroun Hospital at the beginning of the crisis. Besides, the Union of Koura municipalities has a crucial role in providing support and guidance to the municipality.

Initiatives taken by Amioun municipality

On the overall experience, official expressed, “We are doing better than the overall situation in Lebanon”. Several facilitators enabled Amioun municipality to bravely face the pandemic. Since the beginning of the crisis, official retrieved all the updates and evidence-based recommendations from a doctor who works with the World Health Organization (WHO). A focused action plan was executed, including disinfection of streets and trash cans, sorting garbage of COVID-19 people, packing it in special bags and treating it separately. Awareness campaign had been launched to urge people to put masks and adhere to social distancing. This was coupled by financial contributions, in which the municipality distributed food rations, did PCR testing for 90% of the cases, and provided COVID-19 medications for the residents. The municipality also delivered the medications to people’s houses. Most importantly, the municipality managed most COVID-19 cases and followed them at home with the assistance of an active and experienced nurse. It also secured thirteen oxygen respirators to manage critical patients. Alongside, official revealed, “We did trainings to municipal police on how to take full precautions when they accompany stakeholder1”. He added, ‘the municipal police were very strict they were forcing all people in the streets to put masks, restricting the number of people in their vehicles. At the same time, we allowed people to hang out in open areas while putting the mask and ensuring social distancing” He added that people are educated, and this facilitated the process. In this regard, all stakeholders agreed the level of adherence is very satisfactory and that the number of cases dropped significantly since November 2020. Finally, official impressively disclosed, *“We are working on privately buying vaccines to provide it to our community on our expense. We already collected data on people’s ages”*. In addition, the union of municipalities isolated all expats in a hotel and followed them up regularly. According the to stakeholder1, the union of municipalities responded promptly and effectively during the crisis, and it had the upper hand in prompting municipalities to work effectively.

*Barriers facing the municipality*

Five major themes were identified based on the interviews*: lack of awareness and health-consciousness, dereliction of the government, political interference in municipal work, discordance between municipal parties*

On the lack of awareness and absence of health consciousness, stakeholder2 emphasized, “Some cases got infected, and they don’t know that they are critically ill, without the municipal check-ups they would die at home”. Similarly, stakeholder1 cited an incidence that happened with her, *“I had a COVID-19 case of low socio-economic status, his children refused to adhere to our recommendations and transfer him to the hospital. They also interfered in his medications and the oxygen requirement when he was at home. Now the patient is hospitalized, and he is critically ill”.* She also highlighted that self-medication is spread in the community and should be addressed.

The official also added that the municipality was able to establish an isolation center, but it was not sustained because of political interference. The official also mentioned that they worked independently despite the absence of any support from the government. Likewise, stakeholder2 stressed the dereliction of the social forces in implementing the law. He added, *“They see people breaching the law and walking during lockdowns without taking any measure”*. He also stated that the municipality should seek another source of funding other than the government to pursue its role.

On the other hand, stakeholder1 also voiced that official is under scrutiny and his opponents always criticize and underscore his achievements. On the healthcare system, official revealed that some private hospitals were cooperant while others were not. Alongside, stakeholder1 lamented the media of not giving enough credits to the healthcare sector.

| Municipality name: Amioun | | District: Koura | | Governorate: North |
| --- | --- | --- | --- | --- |
| Stakeholders: Official, stakeholder1, stakeholder2 | | | | |
| *Facilitators* | ***Barriers*** | | ***Outcomes*** | |
| Prompt setting of a comprehensive sustainable preparedness plan | Psychological stress | | - - - Fulfilling of basic mandates (raising awareness on social media and through guided tours, distributing masks, hand sanitizers, sterilizing public areas, sorting garbage) - Good isolation and follow up of expats - Supply of medical equipment (13 oxygen respirators, oximeters) - Provision of home-care management of cases by qualified nurses - Free provision of COVID_19 and chronic medications - Free PCR testing for more than 90% of cases - Designating fully equipped ambulances - Private purchase of vaccines to increase the immunization level | |
| Multidisciplinary crisis cell | Limited number of municipal police | |  |  |
| Sufficient human volunteers with rich capacities | Average level of adherence to preventive measures | |  |  |
| Increased donations from immigrants and well-offs in the community | Collapsing economy, shutting down of businesses, and unemployment, and poverty affecting daily-wage earners the most | |  |  |
| Heeded support from the union of municipalities | Discordance and underscoring of municipal efforts by his opponents | |  |  |
| Enhanced equipment of public hospitals | Unsustainable isolation center | |  |  |
| Heightened sense of social coherence and empathy | Delayed reporting of cases | |  |  |
| Credible sources of information during the pandemic |  | |  |  |

Table. Facilitators, Barriers, and Outcome of Amioun Municipality
