# Supplementary material for: Epidemics and local governments in struggling nations: COVID-19 in Lebanon
Source: PLoS One. 2022 Jan 27;17(1):e0262048. doi: 10.1371/journal.pone.0262048 (PMC8794115; doi:10.1371/journal.pone.0262048)
Supplement: S5 File — (DOCX) [file pone.0262048.s006.docx]

**Khiyam Municipality**

- **Brief insight on Khiyam village**

Khiyam is a large village situated in Marjeyoun of Nabatiyeh Governorate of Southern Lebanon, 5 km away from Israeli border. The total number of populations in this village is around 11000 inhabitants, with around 5000 of them are of Syrian nationality. Khiyam, which witnessed major battles between Hezbollah and Israeli Army, became known for the “Khiyam Detention Center” which was operated by the Israeli during their occupation to the Southern of Lebanon. Khiyam is characterized by its agriculture, with more than 50% of the land is used for cultivation of olives, mixed fruits, grains, and vegetables. As for the available facilities, they range from health institutions such as Amel association, Health Ministry clinics to several local institutions that deal with social matters, education, and development (Khiamvillageprofile)

- **Data Collection Process**

Three stakeholders were interviewed: official, stakeholder1, and stakeholder2. To access this municipality, I accounted on a web of connections, namely my academic advisor and a family member who connected me with key informants that facilitated arranging appointments with the key stakeholders. During this period, I was following the Khiam Facebook page given that most municipalities are active on FB during the pandemic. I was also searching in the media, the IMPACT platforms, and MOPH daily report to collect more data about this municipality. Each interview took between 30 and 40 minutes.

- **Khiyam amid COVID-19 pandemic**

*Preparedness of Khiyam municipality*

The municipality took proactive measures before the pandemic reached Lebanon. The municipal council met with different stakeholders (school supervisors, medical teams, Islamic Medical Society, parties, *Mokhtar,* local and international organizations, scouts, and civil society) and created a crisis cell that is very diverse and included representatives of media, finance, and statistics. Then, they distributed tasks among different teams to avoid dereliction. They called for volunteers and they commenced allocating funds for this pandemic. The municipality did a vulnerability assessment to allocate disadvantaged people in the village and classify them into categories (A<B<C), with A being poor, B becoming disadvantaged, and C don’t own homes. Shopkeepers and organizations which were affected by the lockdown measures were also counted for support. The cumulative number of COVID-19 cases in Khiyam municipality as of the date of writing the report (9-4-2021) is 482. The official commenting on the numbers, “ Lebanon is going towards herd immunity’.

*Initiatives taken by Khiyam municipality*

Several actions were taken to curb the spread of the virus in Khiyam village. All expats entering to Khiyam were monitored and isolated after touristic hotels and studios were converted to fully equipped isolation centers. The official added, “We have 17 entries to Khiyam, we kept on 3 major entries that are 24/24 supervised. “*We disinfected cars, took temp for all people on the entries. We allocated volunteers on the most visited places and supermarkets to check people’s temperature and refer suspected individuals for more investigation”.* The official also raised funding from the well-offs in this community and a lot of people donated money, gasoline and food donations. The official proudly proceeded, *“throughout the pandemic the electricity didn’t cut, and the internet was continuously provided to help people cope with the pandemic”.* Besides, they equipped five ambulances and provided medical equipment and other preventive utensils for healthcare workers. Moreover, the municipality, in collaboration with the Islamic Medical Society, allocated a diagnostic center for PCR testing in the village. They also designated a specialized washbasin for covid-19 dead people before recording any death. The official disclosed “it is all about having a powerful administration and management”. In addition, the municipality converted touristic hotels and studios into isolation centers with twenty-two rooms being fully equipped. On the other side, stakeholder1 explained how the municipality responded to the pandemic saying, *“In the first wave, there was some kind of slackening, and the initiatives taken by the municipality were restricted to distributing in kind donations and raising awareness because there was a feeling of victory against covid-19. However, in the second wave, after thousands of cases have surfaced, the municipalities stepped up and felt more concerned and held bigger responsibilities. He added, “the municipality is active, and it did a good work during the pandemic, but the municipality cannot handle everything alone”.*

*Barriers faced by Khiyam municipality*

Six major barriers were mentioned by the stakeholders including: *Stigma and lack of awareness, lack of adherence, scarcity in resources, collapsing economy and prevailing poverty, impeded accessibility to healthcare services, political interference in the municipal work.*

Although the municipality undertook prominent initiatives to combat this pandemic, several obstacles remain in place. The official reported that people were not adhering to lockdown decisions and the municipality called for the security forces to control the chaos. stakeholder1 conveyed that people living in rural areas are buckled under extreme poverty, they do not fear COVID-19 virus, and they rely on God’s fate. Similarly, stakeholder2 reiterated that stigma was prevailing at the beginning of the pandemic, but with time the level of awareness improved gradually. In addition, patients got frustrated when their names were announced on social media platforms because people used to escape them even after recovery.

Another barrier that hampered people’s adherence is the collapsing economy which made things worse. Industries closed, people left their jobs, and more than fifty families moved from Beirut to Khiyam because of increased poverty. The official added that people returned to agriculture and self-sufficiency jobs. This was aggravated by the political unrest and the social dissociation, the official communicated. Upon easing down the restrictions, things started to get out of control because people cannot tolerate more lockdowns in the absence of any viable alternatives, stakeholder2 conveyed.

The official also pinpointed that all hospitals were overwhelmed, and he found difficulty in managing critical COVID-19 cases given the unavailability of beds and all hospitals are far-distanced. On the other side of the coin, “*there was no standardized national plan to be adopted by all municipalities at the national level. We cannot blame the municipalities for developing their own plan, it is the responsibility of the government*”, stakeholder1 commented. He presumed, “*I can’t believe that only 67 out of 314 hospitals are assigned to receive COVID-19 patients at the time where Italy employed 90% of its private hospitals during the pandemic”*.

On the other hand, stakeholder1 reported on the major barriers facing municipalities saying, *“Unfortunately, the municipalities are still buckling under the political inducements which are infringing the developmental mission of municipalities and politicizing them”*. On the other hand, the official complained of the scant resources, “Some municipalities are bankrupt and might shut down at any point of time”

| Municipality name: Khiyam | | District: Marjeyoun | | Governorate: Nabatiyeh |
| --- | --- | --- | --- | --- |
| Stakeholders: Official, stakeholder1, stakeholder2 | | | | |
| *Facilitators* | ***Barriers*** | | ***Outcomes*** | |
| Prompt setting of a comprehensive preparedness plan prior to COVID-19 arrival to Lebanon | Stigma and fear from COVID-19 patients | | - - - Fulfilling of basic mandates (raising awareness on social media and through guided tours, distributing masks, hand sanitizers, sterilizing homes, mosques, and shops) - Distribution of cash and in-kind donations to houses of all infected patients with financial difficulties - Allocating response teams at village entries and frequently visited places - Supply of medical equipment (oxygen respirators, oximeters) - Provision of home-care management of cases by qualified nurses - Establishing a diagnostic center for PCR testing - Provision of psychological, social, and occupational therapies to elderly people - Allocating funds for economically disadvantaged people - Designating five fully equipped ambulances - Provision of electricity and internet 24/24 | |
| Multidisciplinary crisis cell (municipal members, civil society, NGOs, parties, scouts, physicians, medical societies, industries) | Psychological and mental health impacts | |  |  |
| Converting hotels into isolation centers | Lack of adherence to preventive measures and reliance on faith | |  |  |
| Vulnerability assessment | Inaccuracy, delays, and flaws in MOPH surveillance system | |  |  |
| Sufficient human volunteers with rich capacities | Political inducements dominating the municipal authority | |  |  |
| Capability building and training of human resources | Collapsing economy, shutting down of businesses, increased unemployment rate, and prevailing poverty | |  |  |
| Increased donations from immigrants and well-offs in the community | Scarcity in financial resources and capacities | |  |  |
| Creating their own reporting database to overcome reporting flaws | Overwhelmed healthcare system, dearth in equipped COVID_19 hospitals, and insufficient number of beds | |  |  |
| Effective contribution of PHCs | Decreased social ties | |  |  |

Table. Facilitators, Barriers, and Outcomes of Khiyam Municipality
