# Supplementary material for: Epidemics and local governments in struggling nations: COVID-19 in Lebanon
Source: PLoS One. 2022 Jan 27;17(1):e0262048. doi: 10.1371/journal.pone.0262048 (PMC8794115; doi:10.1371/journal.pone.0262048)
Supplement: S6 File — (PDF) [file pone.0262048.s007.pdf]

Roadmap to enhance the response of municipalities during crisis based on the results of the ecological framework

A. At the individual level

- Strengthen the capacity of municipalities through appointing competent and qualified members (Zon, Pavlova, & Groot, 2019).
- Replace structurally bureaucratic management with pragmatic community-based and scientifically practice (Torri et al., 2020).
- Foster cooperation across municipalities through the union of municipalities to promote coherent response, reduce competition on resources, and minimize fragmentation of decisions during a crisis (CDC, 2019; OECD, 2020)
- Provide continuous education and capacity building to municipal police, health and social workers, volunteers, activists, and professionals to support the municipality in implementing measures (Laverack, 2017)
- Assign healthcare specialists in municipal councils (Zon, Pavlova, & Groot, 2019).

B. At the organizational level

- Strengthen the health system functions through improving the active surveillance, laboratory capacities, monitoring and evaluation, and risk communication (Hanvoravongchai et al., 2010).
- Optimize health system capacity through investing in health workforce and healthcare infrastructure in rural areas (Travis et al., 2004).
- Establish and invest in primary care access points in rural areas to improve access to healthcare services (Weeks et al., 2004; Zon, Pavlova, & Groot, 2019).
- Provide supportive environment and employ retention strategies to healthcare professionals (Torri et al., 2020)
- Foster coordination between the public and private healthcare sectors (Hanvoravongchai et al., 2010).
- Carry out seroprevalence testing to determine the extent of outbreaks, identify hotspots and inform public health interventions (Peeling & Olliaro, 2020).

C. At the community level

- Adopt a "Whole-of -society" approach that calls for multi-sector engagement, task sharing, and resource sharing (WHO, 2020).
- Build strong alliances with experts, activists, religious leaders, local organizations, community leaders, schools, hospitals, and primary healthcare centers to foster better response (communicating messages, providing spiritual and social support, sharing resources) (CDC, 2020; Public Health Agency of Canada, 2020)
- Engage religious leaders in decision-making and in risk communication to avoid rebellion against national policies aiming at preventing religious gatherings (WHO, 2020b)

- Replace social and religious gatherings with virtual alternatives, otherwise strict measures should be implemented to mitigate the risk of viral transmission (WHO, 2020b)
- Leverage on community-level programs to assist COVID-19 patients and their families with their functional needs (i.e., food, medication) to maintain proper isolation at home (CDC, 2019).
- Proactively communicate timely and accurate information within the community using the right channels and test whether messages are well received (CDC, 2019).
- Identify fake news and misinformation and demystify uncertain information to the public using effective communication (Abu Samra & El-Jardali, 2020)
- Encounter stigma and stereotyping through raising awareness and addressing the discriminatory behaviors without increasing fear (CDC, 2019).
- Undertake community and healthcare-based approaches to reduce stigma such as generating solidarity, fostering empathy, and providing institutional support (Logie, 2020).
- Coordinate with the Ministry of Social Affairs and concerned organizations to provide mental and psychosocial support to COVID-19 patients and their families (Abu Samra & El-Jardali, 2020)
- Develop culturally relevant mental health programs that address stigma associated with the pandemic (Maulik, Thornicroft, Saxena, 2020).

D. At the policy level

- Enforce multi-level coordination between national and subnational government representatives to minimize the risk of a fragmented crisis response
- Set and implement allocation criteria that is guided by strategic regional priorities to minimize inequitable distribution of resources among different localities
- Advocate for the enactment of the de-centralization law to grant the local governments a wide range of political, financial, and administrative power in condition that accountability mechanisms are put into place at both local and national levels (Crook, 2003, Isufaj, 2014).
- Allocate special grant schemes for municipalities during crisis to avoid unfunded mandates and to minimize the gaps between the increasing expenditures and decreasing revenues during the pandemic.
- Develop and implement evidence-based policies to address mental health needs of affected people and involve the teams that are managing COVID-19 patients (Maulik, Thornicroft, Saxena, 2020).
