# Supplementary material for: Epidemics and local governments in struggling nations: COVID-19 in Lebanon
Source: PLoS One. 2022 Jan 27;17(1):e0262048. doi: 10.1371/journal.pone.0262048 (PMC8794115; doi:10.1371/journal.pone.0262048)
Supplement: Attachment — Submitted filename: Response to Reviewers (municipalities).docx [file pone.0262048.s008.docx]

**Response to Reviewers**

**Academic Editor:**

1. Please ensure that your manuscript meets PLOS ONE's style requirements, including those for file naming.

The manuscript is adjusted according to PlOS One guidelines.

2. Please amend your current ethics statement to address the following concerns:  
a) Did participants provide their written or verbal informed consent to participate in this study? 
b) If consent was verbal, please explain i) why written consent was not obtained, ii) how you documented participant consent, and iii) whether the ethics committees/IRB approved this consent procedure.

The ethics statement was amended as requested.

3. 3. When reporting the results of qualitative research, we suggest consulting the COREQ guidelines  or other relevant checklists listed by the Equator Network, such as the SRQR, to ensure complete reporting

The manuscript was checked against the COREQ guideline and complete reporting was ensured.

4. We note that you have stated that you will provide repository information for your data at acceptance. Should your manuscript be accepted for publication, we will hold it until you provide the relevant accession numbers or DOIs necessary to access your data.

Reserved DOI: 10.17632/k4jp3xscg2.1

5. We note that Figure 1 in your submission contain map images which may be copyrighted. All PLOS content is published under the Creative Commons Attribution License (CC BY 4.0)

Permission to use the map is included in “other” supplement.

6. We note you have included a table to which you do not refer in the text of your manuscript. Please ensure that you refer to Table 2 in your text;

Table 2 was cited

7. Please include your tables as part of your main manuscript and remove the individual files. Please note that supplementary tables (should remain/ be uploaded) as separate "supporting information" files.

Tables were added to the manuscript.

8. Please include captions for your Supporting Information files at the end of your manuscript, and update any in-text citations to match accordingly.

Captions were added.

**Reviewer 1:**

1. I would strongly encourage respondents to either anonymise the names of all the municipalities or describe roles in more generic terms, for example “Official in municipality”.

All identifiers were replaced with generic terms (eg, officials, stakeholders, key informants)

2. a. The time period when the data were collected (i.e. interviews were conducted) is not clear

The time period of the conducted interviews was added in the study procedures section.

b. The research was carried out in nine municipalities, however the authors restrict their analysis to four municipalities. It would be helpful to explain the rationale for this.

The rational for including 4 municipalities in this paper is to keep it short, concise, and readable (added this to the plan of analysis)

c. One of the key discussion points relates to the proximity of municipalities from the capital. However, this is not described in sufficient detail in the results. It would be helpful to know, for example, whether both of the municipalities with adequate response were closer to the capital than those with inadequate response. The map may be helpful in this respect if it showed where capital was located on the map.

The response of the municipalities to the capital in regard to their response is more pronounced for the 9 municipalities, with most of the proximal municipalities responded better to the pandemic due to the availability of resources and better healthcare systems. However, I removed the sentence that relates proximity to the response because on of the selected municipalities which responded well to the pandemic is not proximal to the capital (khiyam).

d. Some of the statements in your discussion could have benefitted from references. For example, the entire discussion about the inequitable distribution of resources (on page 26) is not supported by existing literature.

References are added

Finally, here are some miscellaneous issues for the authors to consider:
a. “eternally” was removed
b. The ignorance of religious leaders was modified to “ignorance in health issues”.

**Reviewer 2:** 
1) I opted not to include percentages because in this qualitative study, we are not interested in quantification, rather we want to get in depth insights on the experiences of various municipalities during the pandemic. If I put percentage in the abstract, then I must add percentages to all the results.

2) (page 8) Please elaborate on "the felt presence to the virtually absent". It is difficult to understanding the meaning. Some municipalities were distinguished by the activities they undertook, thus making their presence noticed by people, while other municipalities get unnoticed because they were not active during the pandemic

3) (page 8) Please justify why SEM was used rather than other frameworks. And outline advantages and disadvantage

I used SEM because it is an ideal tool to address complex public health issues, as it allows deep understanding of the dynamic interplay between personal, interpersonal, organization, community and policy levels. In other words, we can see the multi-level barriers that impeded municipalities from responding well to the pandemic and design interventions at different levels (individual, community, policy…) to overcome these barriers. However, one of the major imitations of SEM is that the programs can be expensive to implement. In addition, it requires close coordination between individuals and groups for effective implementation.

4) Methods (p1ge 11) Could you provide the IRB number?

IRB number: SBS-2020-0428

5) The results and discussion sections are lengthy, making it challenging for the reader to focus on the core messages. Please try to modify/shorten the text to make it more coherent and succinct

I shortened the results and discussion as much I can, but this study has a lot of themes and subthemes that cannot be omitted or overlooked.

6) Please carefully check for typos and correct grammar e.g. (page 8 SEM has been adapted

The paper was spell checked and corrected.
